# Health symptoms and post-COVID-19: Comparing symptomatic groups based on self-reported and primary care data

**DOI:** 10.1371/journal.pone.0323960

**Published:** 2025-06-12

**Authors:** Jenny Gerbecks, C. Joris Yzermans, Michel L. A. Dückers, Mark Bosmans, Christos Baliatsas

**Affiliations:** 1 Netherlands Institute for Health Services Research (Nivel), Utrecht, the Netherlands; 2 University of Groningen (UG), Groningen, the Netherlands; Universitair Kinderziekenhuis Koningin Fabiola: Hopital Universitaire des Enfants Reine Fabiola, BELGIUM

## Abstract

**Introduction:**

Health symptoms are common in the general population. Relatively little research has focused on health symptoms in context of the COVID-19 pandemic between people with different manifestations of COVID-19. Aim of this study was to assess symptom differences between individuals suffering from post-COVID-19, individuals infected with COVID-19 but not suffering from lasting symptoms (‘ex-covid’), and non-infected individuals.

**Methods:**

A 2022 nation-wide cross-sectional health survey was combined with routine primary care data. The response rate for the survey was 32%. The questionnaire data consisted of 315,586 respondents, and the electronic health record (EHR) data included 29,797 patients with merged questionnaire data. Prevalence of individual symptoms and number, duration, and severity of symptoms were analyzed.

**Results:**

Individuals with post-COVID-19 reported more (IRR 1.48 [CI 1.46–1.49]), longer lasting (1.92 [CI 1.88–1.96]), and more severe symptoms (2.00 [CI 1.96–2.05]) than the ex-covid group. Post-COVID-19 also reported more (1.55 [CI 1.52–1.57]), longer lasting (1.87 [CI 1.82–1.92]), and more severe symptoms (1.95 [CI 1.89–2.01]) compared to non-infected. Ex-covid reported more symptoms than the non-infected, but on average, their symptoms lasted a shorter duration and were experienced as less severe. In EHRs, symptoms between groups showed generally the same pattern.

**Conclusion & discussion:**

This study points at variation in symptomatology after COVID-19 infection. Individuals with post-COVID-19 experienced more, longer-lasting, and more severe symptoms compared to the other two groups. This study was one of the first to assess group differences between groups with different types of COVID-19 infections.

## Introduction

Health symptoms are common in the general population [1–3], and may persist for a long period of time, affecting daily life [4–6]. They may have an important societal impact and their management is challenging for healthcare, considering that they can be associated with functional difficulties [7,8]. Primarily due to the increased health care utilization it has been estimated that medical expenses associated with health symptoms account for more than 10% of the annual health care expenditure in the working population [9,10].

In March 2020, the WHO declared COVID-19 a global pandemic. The spread of the virus and precautionary measures taken to limit the viral transmission have contributed to the development of physical and mental health issues, while also aggravating existing health problems [11,12]. The Centers for Disease Control and prevention (CDC) reported that beyond the acute phase of infection – which typically included fever, cough, shortness of breath, sore throat, congestion or runny nose, new loss of taste or smell, fatigue, muscle or body aches, headache, nausea or vomiting and/or diarrhea [13], many individuals developed persistent symptoms up to months after the infection, called post-COVID-19 conditions. Post-COVID-19 can include for example fatigue, shortness of breath, and cognitive dysfunction, but is not very well defined: a variety of over 200 symptoms have been reported [14]. These symptoms affect every day functioning and quality of life. Anyone being infected with COVID-19 is at risk of developing post-COVID-19 conditions [15], and an estimated 10–15% of those infected with COVID-19 exhibits post-COVID-19 conditions [16,17]. Up to now, symptom profiles, prevalence, and severity remain poorly understood.

Existing research highlights fatigue, musculoskeletal issues, headache, diarrhea, shortness of breath, memory loss, concentration difficulties and sleeping problems as the most common post-COVID-19 symptoms [18–21]. The plethora of symptoms affecting multiple organ systems suggests the presence of different underlying mechanisms [22]. However, evidence on symptom duration, on perceived seriousness, and on demographic differences is limited.

Previous research thus pinpointed the most frequently reported symptoms, but evidence on the clinical importance on the basis of characteristics, or duration and severity of symptoms, is still scarce. Duration of symptoms is hard to ascertain [16]. Karuna et al. [23] concluded that post-COVID-19 symptom severity was different between countries, insinuating that demographics or culture might influence symptom severity. Sivan et al. [7] concluded that symptom severity is positively associated with functional difficulty scores, and negatively associated with overall health. Most studies focus solely on the post-COVID-19 group, and do not compare this group to those infected without longer-lasting symptoms, and the non-infected. Hampshire et al. [24] studied cognitive impairment symptom differences between infected people with post-COVID-19, and infected without post-COVID-19, and found the two groups differ in symptoms reporting and severity. If this is true for cognitive impairment symptoms, this might also be the case for other symptoms. Also, when studies include post-COVID-19 symptoms, they rely on self-reported assessment and relatively small samples which come with biases and limited power. Population-level studies using more objective data, such as primary care records, are scarce.

This study addresses these gaps by examining symptom prevalence, duration and perceived seriousness using both self-reported questionnaires and general practitioner (GP) data. Three groups were compared: individuals who reported they are suffering from post-COVID-19, those that have been infected with COVID-19 without persistent symptoms, and individuals that, to their knowledge, have never been infected with COVID-19. Our study allows for a broad variety of symptoms to be analyzed in a sizable sample of people. We assess these research questions:

1) Do people with post-COVID-19, or people with a previous COVID-19 infection differ in individual symptoms experienced than non-infected individuals?
2) Do people with post-COVID-19, or people with a previous COVID-19 infection experience more, longer lasting, and more severe symptoms than non-infected individuals?
3) How do self-reported symptoms align with GP-registered symptoms across groups?

## Methods

### Questionnaire data

The Dutch Public Health Monitor Adults and Elderly 2022 (DPHM 2022) is a nationwide survey conducted in the autumn of 2022, to assess the health, well-being, and lifestyle of Dutch residents aged 18 and older. Developed collaboratively by the Community Health Services (GGDs), Statistics Netherlands, and the National Institute for Public Health and the Environment (RIVM), participants were randomly selected from the Personal Records Database (BRP) to represent the Dutch population. Respondents could fill in the questionnaire online or on paper between September and December 2022. One reminder was sent to non-responders. The Amsterdam Medical Center declared this study non-WMO obligated. This means it is not liable to further ethical review because it is not subject to the Dutch Medical Research Involving Human Subjects Act [25]. Data were handled confidentially, ensuring anonymity through group-level reporting and a data protection impact assessment. By filling out the questionnaire, participants consented to use of their data. Data collection for the DPHM 2022 occurred to a random sampling process, performed by Statistics Netherlands. Please see Van Duinkerken et al. [26] for more information on the sampling process.

The “Symptoms and Perceptions” (SaP) questionnaire [27] was used to assess self-reported symptoms. This questionnaire includes 28 symptoms in different organ systems, that may also be registered by a general practitioner, using the ICPC (see below), when a patient consults the GP because of this symptom. For this particular version of the DPHM, two COVID-19-specific symptoms were included (sore throat, and loss of smell or taste). Respondents reported symptoms experienced in the past month, their duration(less than 1 month, 1–6 months, or longer than 6 months), and whether they consulted a general practitioner (GP) for this symptom. Three symptom scores were calculated: number of symptoms, duration of symptoms, and symptom severity. Number of symptoms indicates the number of symptoms the respondent reported based on the SaP questionnaire. Duration of symptoms is not linear, but a higher score on this variable indicates a longer duration of the combined experienced SaP symptoms. Symptom severity indicates whether or not the respondent visited the general practitioner as a measure of the perceived seriousness of the symptom.

### General practitioner data

In the Netherlands, every inhabitant is obligatory enlisted in just one general practice. Thus, no one is omitted from health records. GPs serve as gatekeeper to specialized care, and systematically record health data in electronic health records (EHRs) using the International Classification of Primary Care (ICPC) [28]. We used data from the Nivel Primary Care Database, which consists of routinely recorded data from health care providers in a representative sample of the Dutch population [29]. Regarding the use of primary care data, the present study does not fall within the scope of the Medical Research Involving Human Subjects Act and therefore did not require ethical approval. The use of electronic health records for research purposes is allowed under certain conditions. When these conditions are fulfilled, approval by a medical ethics committee is not obligatory for this type of observational studies containing no directly identifiable data (art. 24 GDPR implementation Act jo art. 9.2 sub j GDPR). The privacy aspects of data utilization were approved by the Privacy Commission of Nivel.

For this study, we used the ICPC symptoms that correspond with COVID-19 and SaP symptoms that were available in the GP data. For a subset of the total questionnaire survey population, it was possible to merge self-reported data from the DPHM with available EHRs from general practices in the Nivel Primary Care Database (PCD). This merging was accomplished using unique pseudonymized identifiers that allowed for secure linking of the two datasets. The EHR data was uploaded to the secure digital environment of the Dutch Central Bureau of Statistics/Statistics Netherlands (CBS), where the survey data are also housed. This led to a dataset with almost 30,000 individuals with both their electronic health record data and their individual answers to the GGD survey.

### Definition of symptomatic groups

We distinguish between infected people with post-COVID-19 and infected without post-COVID-19 mainly because previous research [24] has found that the two groups can differ in symptom reporting and severity. The full survey dataset was categorized into three groups;

1. *Post-covid*: individuals infected with COVID-19 over three months ago and still experiencing symptoms attributed to the infection;
2. *Ex-covid*; individuals who were infected, but did not report persistent symptoms;
3. *Non-infected*: those who reported never being infected, serving as the base group; a reference group with no effects from the COVID-19 virus whatsoever.

### Events during the pandemic

The DPHM 2022 included a question on experiencing various COVID-19-related events. Life events, or other impactful events, might induce health symptoms, independently of socio-economic status, symptoms of anxiety and depression, and sex [30]. Events related to the COVID-19 pandemic in particular, might put extra stress on the individual in already stressful times. In this study, we want to rule out a possible role of pandemic life events in the prevalence of health symptoms. For this study, we included four events: ‘’I personally experienced hospitalization due to COVID-19,” ‘’someone significant to me was hospitalized due to COVID-19,” ‘’someone significant to me passed away due to COVID-19,” and ‘’due to the COVID-19 social distancing measures, I could not say goodbye to someone significant to me.” These pandemic events analyses are included as sensitivity analyses.

### Statistical analyses

Three types of regression analyses were conducted; logistic regression analyses for individual SaP-symptoms, negative binomial regression analyses for SaP symptom scores, and mixed effects logistic multilevel regression analyses for GP data analyses. All analyses were adjusted for age (linear), gender (dichotomous), income (categorical, in quintiles), educational level (categorical), ethnic background (categorical), obesity (dichotomous), smoking behaviour (categorical), and excessive use of alcohol (dichotomous). The GP analyses were also adjusted for part of the year registered at the general practice (categorical, in quartiles), and general practices were listed at the second level. The results for the confounders are discussed briefly in the main text, but are not presented in the main tables. These results can be found in S1 - S3 Tables in S1 File. Incidence rate ratios (IRR) or odds ratios (OR), and 99% (questionnaire sample) or 95% (GP sample) confidence intervals (CI) were calculated. Analyses were performed in STATA version 16.1 (StataCorp LP, College Station, TX, USA). “False Discovery Rate” (FDR) correction [31,32] was applied for multiple comparisons.

## Results

### Questionnaire

The response rate was 32%. The final study sample consists of 315,586 individuals. Demographic characteristics for the questionnaire and GP sample are provided in Tables 1 and 2.

**Table 1:** Demographic characteristics per group, questionnaire sample.

	Post-covid group		Ex-covid group		Non-infected group	
	N = 18,416		N = 158,309		N = 138,861	
*Demographic characteristics (%)*						
Mean age (SD)	55.4	(16.5)	54.7	(17.7)	65.75	(15.5)
Female gender	10,893	(59.2)	86,829	(54.9)	72,016	(51.9)
Income						
- quintile 1	1,795	(9.8)	10,781	(6.9)	15,309	(11.1)
- quintile 2	3,147	(17.2)	21,764	(13.9)	31,376	(22.8)
- quintile 3	4,030	(22.0)	32,018	(20.4)	31,516	(22.9)
- quintile 4	4,745	(25.9)	41,928	(26.7)	30,524	(22.2)
- quintile 5	4,605	(25.1)	50,496	(32.2)	29,026	(21.1)
Education						
- lower	5,562	(30.4)	39,614	(25.2)	56,815	(41.3)
- middle	6,726	(36.8)	50,937	(32.3)	28,919	(28.3)
- higher	6,008	(32.8)	66,982	(42.5)	41,761	(30.4)
Foreign ethnic background (%)						
- autochthonous	15,589	(84.7)	137,708	(87.0)	119,568	(86.1)
- European migrant	1,103	(6.0)	9,032	(5.7)	8,569	(6.2)
- non-European migrant	1,724	(9.4)	11,569	(7.3)	10,723	(7.7)
*Lifestyle characteristics (%)*						
Obesity	4,273	(23.7)	22,414	(14.4)	22,767	(16.9)
Smoking behaviour						
- non-smoker	7,808	(43.3)	73,846	(47.9)	53,491	(39.9)
- ex-smoker	8,155	(45.2)	63,314	(41.0)	62,136	(46.4)
- smoker	2,067	(11.5)	17,167	(11.1)	18,413	(13.7)
Excessive use of alcohol	2,989	(16.6)	29,528	(19.0)	24,380	(18.2)
*SaP symptoms mean (SD)*						
Number of symptoms	11.5	(6.1)	7.6	(5.4)	6.8	(5.3)
Duration of symptoms	5.5	(5.2)	2.7	(3.6)	2.9	(3.8)
Severity of symptoms	3.8	(4.7)	1.8	(2.9)	2.1	(3.2)

**Table 2:** Demographic characteristics per group, GP registered sample.

	Post-covid group		Ex-covid group		Non-infected group	
	1,645		13,930		11,806	
*Demographic characteristics (%)*						
Mean age (SD)	55.94	(16.2)	55.98	(17.6)	65.8	(15.3)
Female gender	975	(59.3)	7,578	(54.4)	6,108	(51.7)
Income						
- quintile 1	151	(9.2)	925	(6.7)	1,283	(10.9)
- quintile 2	267	(16.3)	2,001	(14.4)	2,730	(23.2)
- quintile 3	371	(22.6)	2,908	(21)	2,711	(23.1)
- quintile 4	409	(25.0)	3,764	(27.2)	2,625	(22.3)
- quintile 5	441	(26.9)	4,263	(30.8)	2,407	(20.5)
Education						
- lower	512	(31.3)	3,526	(25.4)	4,885	(41.8)
- middle	608	(37.2)	4,599	(33.2)	3,358	(28.7)
- higher	516	(31.5)	5,736	(41.4)	3,458	(29.6)
Foreign ethnic background						
- autochthonous	1,406	(85.5)	12,344	(88.6)	10,298	(87.2)
- European migrant	105	(6.4)	759	(5.5)	742	(6.3)
- non-European migrant	134	(8.2)	827	(5.9)	766	(6.5)
*Lifestyle characteristics (%)*						
Obesity	373	(23.2)	1,956	(14.3)	1,955	(17)
Smoking behaviour						
- non-smoker	672	(41.8)	6,396	(47.3)	4,559	(40.1)
- ex-smoker	760	(47.3)	5,639	(41.7)	5,241	(46.1)
- smoker	174	(10.8)	1,501	(11.1)	1,569	(13.8)
Excessive use of alcohol	275	(17.2)	2,535	(18.6)	2,076	(18.2)

Average age in the Dutch general population in 2022 was 43.3 years, whereas for our sample this is 59.6 years, indicating that older people are overrepresented. In our sample, this is In both the questionnaire and the GP sample, those with post-COVID-19 are relatively often overweight. Lower educated seem to be a bit overrepresented in our sample, however, the categories are hard to compare to the categories from StatLine [33]. Compared to the general population, in 2022, 13.3% of the general Dutch population was overweight with a BMI ≥ 30 [34]. Furthermore, StatLine did provide information on nationalities, but not on ethnic background per se. Because someone with a migrational background can still have the Dutch nationality, these numbers are hard to compare.

**Fig 1:**
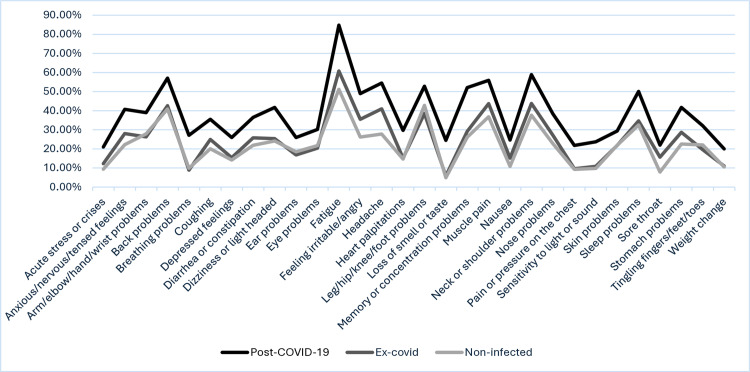
Prevalence of SaP symptom per group, questionnaire data.

Fig 1 illustrates the prevalence of SaP across groups in the questionnaire sample. Symptom prevalence is highest in the post-covid group. Generally, prevalence of symptoms is higher among ex-covid compared to non-infected, except for eye irritation, ear symptoms, arm/elbow/hand/wrist symptoms, leg/hip/knee/foot symptoms, tingling of fingers, feet or toes, and shortness of breath or wheezing. These differences between ex-covid and non-infected are notably smaller than the differences between post-covid and ex-covid.

Table 3 presents odds ratios for reporting SaP symptoms across groups: post-covid vs. ex-covid, post-covid vs. non-infected, and ex-covid vs. non-infected. The post-covid group is at higher risk of all the health problems than both the non-infected and ex-covid. The ORs range from 1.49 higher chance (skin problems), to 8.54 (loss of smell or taste). Comparatively, the ex-covid group shows smaller differences from the non-infected group, with significant ORs ranging from 0.90 (depressed feelings) to 1.55 (loss of smell or taste). We also compared the post-covid group to the ex-covid group. Individuals suffering from post-COVID-19 report significantly more symptoms. We classified symptoms with ORs above 2.00 as typical post-COVID-19 symptoms.

**Table 3:** Group differences for on the individual SaP symptoms (Odds Ratios, CI 99%)^a^.

	Post-covid vs. Ex-covid		Post-covid vs. Non-infected		Ex-covid vs. Non-infected	
	OR	CI	OR	CI	OR	CI
Abdominal/stomach pain	**1.76**	**(1.68–1.84)**	**1.88**	**(1.79–1.97)**	**1.05**	**(1.03–1.08)**
Acute (intense) stress or crisis	**1.91**	**(1.81–2.02)**	**1.83**	**(1.72–1.93)**	**.95**	**(.91–.98)**
Arm/elbow/hand/wrist symptoms	**1.65**	**(1.58–1.72)**	**1.66**	**(1.59–1.74)**	**1.04**	**(1.01–1.06)**
Back problems	**1.68**	**(1.61–1.75)**	**1.72**	**(1.65–1.80)**	**1.03**	**(1.00–1.05)**
Cough	**1.63**	**(1.56–1.70)**	**2.12**	**(2.02–2.22)**	**1.28**	**(1.25–1.31)**
Diarrhea	**1.60**	**(1.53–1.67)**	**1.69**	**(1.61–1.77)**	**1.03**	**(1.00–1.05)**
Dizziness or feeling light-headed	**2.02**	**(1.94–2.11)**	**2.07**	**(1.98–2.17)**	**1.00**	**(.98–1.02)**
Ear symptoms	**1.70**	**(1.62–1.78)**	**1.78**	**(1.69–1.89)**	**1.03**	**(1.01–1.06)**
Eye irritation	**1.61**	**(1.54–1.69)**	**1.65**	**(1.57–1.73)**	**1.00**	**(.98–1.03)**
Fatigue/tiredness	**3.69**	**(3.48–3.91)**	**4.21**	**(3.97–4.47)**	**1.12**	**(1.10–1.15)**
Feeling anxious/nervous/tense	**1.83**	**(1.74–1.91)**	**1.87**	**(1.78–1.95)**	**1.01**	**(.99–1.04)**
Feeling down/depressed	**1.89**	**(1.80–1.99)**	**1.74**	**(1.65–1.83)**	**.90**	**(.88–.93)**
Feeling irritable/angry	**1.81**	**(1.74–1.89)**	**1.99**	**(1.90–2.08)**	**1.10**	**(1.07–1.12)**
Headache	**1.86**	**(1.78–1.95)**	**2.11**	**(2.01–2.21)**	**1.14**	**(1.11–1.17)**
Heart palpitations	**2.24**	**(2.14–2.35)**	**2.30**	**(2.18–2.41)**	**1.03**	**(1.00–1.06)**
Hypersensitivity to light or sound	**2.48**	**(2.35–2.61)**	**2.28**	**(2.16–2.41)**	**.93**	**(.90–.96)**
Leg/hip/knee/foot symptoms	**1.64**	**(1.57–1.71)**	**1.68**	**(1.61–1.76)**	**1.04**	**(1.02–1.06)**
Loss of smell or taste	**5.08**	**(4.81–5.37)**	**8.54**	**(8.02–9.09)**	**1.55**	**(1.48–1.63)**
Memory or concentration problems	**2.61**	**(2.50–2.73)**	**2.76**	**(2.64–2.89)**	**1.05**	**(1.02–1.07)**
Nasal symptoms	**1.65**	**(1.58–1.72)**	**1.98**	**(1.90–2.08)**	**1.17**	**(1.14–1.20)**
Nausea	**1.82**	**(1.73–1.92)**	**1.83**	**(1.73–1.93)**	**.99**	**(.96–1.02)**
Neck or shoulder symptoms	**1.78**	**(1.70–1.86)**	**1.96**	**(1.87–2.05)**	**1.11**	**(1.09–1.13)**
Pain in muscles	**1.63**	**(1.56–1.70)**	**1.80**	**(1.72–1.88)**	**1.09**	**(1.06–1.11)**
Pain or pressure in chest	**2.52**	**(2.38–2.65)**	**2.49**	**(2.36–2.64)**	**.99**	**(.95–1.02)**
Shortness of breath or wheezing	**3.51**	**(3.33–3.69)**	**3.37**	**(3.20–3.56)**	**.94**	**(.91–.98)**
Skin symptoms	**1.44**	**(1.37–1.51)**	**1.49**	**(1.42–1.56)**	**1.01**	**(.99–1.04)**
Sleep problems	**1.82**	**(1.74–1.90)**	**1.81**	**(1.73–1.89)**	**1.03**	**(1.00–1.05)**
Sore throat	**1.59**	**(1.51–1.67)**	**2.33**	**(2.20–2.46)**	**1.48**	**(1.42–1.53)**
Tingling fingers/feet/toes	**1.79**	**(1.71–1.88)**	**1.75**	**(1.66–1.83)**	**.98**	**(.96–1.01)**
Weight change	**1.81**	**(1.71–1.91)**	**1.72**	**(1.62–1.82)**	**.93**	**(.90–.96)**

^a^Adjusted for age, gender, income, education, migration status, obesity, smoking behaviour, and excessive use of alcohol.

Table 4 shows incidence rate ratios (IRR) for SaP sum scores, with full confounder details available in S1 Tables in S1 File. The post-covid group is at higher risk of experiencing more, longer-lasting, and more perceived seriousness than the non-infected. Women are at greater risk than men, and symptom risk decreases with higher income. Education and migration background show small but statistically significant differences. Smokers and ex-smokers are both at higher risk of symptoms compared to non-smokers. The ex-covid group shows minimal differences with the non-infected group: both have similar risks across all three SaP sum scores.

**Table 4:** Incidence Rate Ratios for group comparison on the SaP symptom variables (CI = 99%)^a^.

	Number of symptoms		Duration of symptoms		Symptom severity	
	IRR	CI	IRR	CI	IRR	CI
Post-covid vs. Ex-covid	**1.48**	**(1.46–1.49)**	**1.92**	**(1.88–1.96)**	**2.00**	**(1.96–2.05)**
Post-covid vs. non-infected	**1.55**	**(1.52–1.57)**	**1.87**	**(1.82–1.92)**	**1.95**	**(1.89–2.01)**
Ex-covid vs. Non-infected	**1.04**	**(1.03–1.05)**	**.98**	**(.96–.99)**	**.98**	**(.97–1.00)**

^a^Adjusted for age, gender, income, education, migration status, obesity, smoking behaviour, excessive use of alcohol, part of the year registered at the general practitioner.

### General practitioner electronic health records

Table 5 shows the GP data (symptoms presented to the GP and/or diagnosed by the GP). Post-COVID-19 patients are at higher risk of presenting fatigue, shortness of breath, feeling depressed, dizziness, and coughing, compared to the ex-covid group. Those with post-covid report more COVID-19-related symptoms to the GP than the non-infected, with significant differences for fatigue, shortness of breath, headache, sleep problems, and dizziness, but not for depressed feelings or memory or concentration problems. Post-COVID-19 patients are more than twice as likely to consult a GP for fatigue and shortness of breath compared to non-infected individuals. In contrast, differences between the ex-covid group and the non-infected are mostly not significant.

**Table 5:** Association between Post-covid, Ex-covid, and Non-infected, and symptoms typical for COVID-19 reported to the general practitioner, significant results (CI 95%) printed in bold.

	Post-covid vs. Ex-covid		Post-covid vs. Non-infected		Ex-covid vs. Non-infected	
	OR	CI	OR	CI	OR	CI
Fatigue	**2.39**	**(1.94–2.93)**	**2.58**	**(2.07–3.20)**	.97	(.86–1.14)
Memory or concentration problems	1.24	(.78–1.98)	1.60	(.99–2.56)	1.18	(.93–1.49)
Shortness of breath	**2.53**	**(1.85–3.45)**	**2.20**	**(1.61–3.01)**	.92	(.74–1.13)
Headache	1.34	(.94–1.90)	**1.53**	**(1.06–2.22)**	1.18	(.96–1.45)
Feeling depressed/down	**1.83**	**(1.27–2.65)**	1.67	(.52–5.35)	.93	(.49–1.75)
Sleep problems	1.29	(.99–1.69)	**1.33**	**(1.01–1.74)**	.98	(.86–1.13)
Dizziness or feeling light- headed	**1.41**	**(1.00–1.99)**	**1.51**	**(1.06–2.14)**	1.02	(.84–1.22)
Nausea	1.60	(.80–3.20)	1.01	(.51–2.00)	**.54**	**(.37–.78)**
Feeling anxious	1.13	(.78–1.63)	1.29	(.87–1.91)	1.13	(.92–1.38)
Acute stress or crisis	1.14	(.71–1.85)	1.08	(.65–1.79)	1.00	(.77–1.32)
Feeling irritable/angry	1.63	(.56–4.78)	1.67	(.52–5.35)	.93	(.49–1.75)
Coughing	**1.38**	**(1.14–1.68)**	**1.53**	**(1.25–1.87)**	1.10	(.99–1.23)
Weight change	.92	(.46–1.85)	.87	(.43–1.76)	.81	(.60–1.11)

^a^Adjusted for age, gender, income, education, migration status, obesity, smoking behaviour, excessive use of alcohol, part of the year registered at the general practitioner.

### Sensitivity analyses

We applied the False Discovery Rate (FDR) (Benjamini and Hochberg method) [31,32] (results not shown), to account for multiple testing on the associations in Tables 3 and 4. Using a conservative q-value of 0.01, all associations between both the post-covid and the ex-covid group, and the post-covid and the non-infected group remained significant. Three associations between the ex-covid and the non-infected group that initially were significant were no longer significant. This affirms the robustness of our findings, especially for the post-COVID-19 comparisons.

A factor analysis of SaP symptoms indicated that most of the items measure a single factor (Cronbach’s alpha 0.86 for both the questionnaire and the EHR sample). Prevalence of symptoms did not differ across regions (results not shown). We also assessed whether differences between the three groups altered when we included four pandemic-related events in the analyses. The results (excluding confounders) are shown in S4 – S6 Tables in S1 File. Some interaction analyses confirmed that pandemic events in combination with COVID-19 exposure sometimes mattered in terms of symptoms experienced.

## Discussion

The large sample size enhances statistical power and robustness of the conclusions, while the inclusion of both self-reported data and GP records allow for unique group comparisons between affected and unaffected groups. This dual approach is rare in (post-)COVID-19 literature, since most studies focus solely on post-COVID-19 symptoms, and not on comparison between affected and non-affected groups [16,18,19,35]. Reliability of the GP data is high, given that they are routinely collected by the Nivel Primary Care Database in a Dutch representative sample, minimizing data collection errors. A limitation may be the self-reported nature of COVID-19 status. Some non-infected respondents may have unknowingly been infected (e.g., asymptomatic cases) [36], and some infections may have been misclassified as COVID-19. However, if there is any effect from this group mix-up, it should have made the differences smaller, and then results from our study are an underestimation. In addition, vaccination status might have affected chances of experiencing symptoms or, in the end, presenting with post-COVID-19. Unfortunately, inclusion of vaccination status was no option for this study. Another limiting factor might be the overrepresentation of older people, compared to the Dutch general population. We chose to not weigh our dataset, since weighting assumes that survey participants within each demographic group can be adjusted to represent the non-respondents from that same group. This assumption is impossible to fully verify, particularly for outcomes such as health symptoms. Our study was not aimed at estimating prevalence in the general population but rather at comparing symptomatology across specific groups.

Post-covid group reported more frequent, longer lasting, and more severe symptoms than both comparison groups. Ex-covid group reported more symptoms than the non-infected, but these were generally of shorter duration and less severe compared to the non-infected. This pattern was consistent across both self-reported and GP data, and regression analyses confirmed that they were not attributable to demographic factors or pandemic stressors. Even though we tested thoroughly and we adjusted for various aspects that in general are related to individuals’ health, differences between the groups remain. With regards to the socio-demographics, the general patterns show that females, lower incomes, migrational background, obesity, (ex-)smokers are at significantly higher risk of more, longer lasting, and more severe symptoms.

Previous studies identified musculoskeletal issues, headache, diarrhea, fatigue, and shortness of breath to be the most frequently reported symptoms among post-COVID-19 patients [18]. Our findings support this and highlight specific symptoms, such as fatigue/tiredness, heart palpitations, pain or pressure in chest, dizziness or light-headed, memory or concentration problems, shortness of breath, hypersensitivity to lights and sounds, and loss of smell or taste as ‘typical’ post-COVID-19 symptoms. GP data corroborates this, identifying fatigue and shortness of breath as typical post-COVID-19 symptoms. These patterns were not influenced by demographic factors or life events, suggesting that they are directly related to the virus. The other symptoms, which are less typical for Post-COVID-19 conditions, are also increased for the post-covid group compared to both the ex-covid and the non-infected group.

Interestingly, memory and concentration issues – often associated with post-COVID-19 – did not differ significantly in GP data between post-covid and non-post-covid groups. This might be due to an increase of memory and concentration problems in the general population during the pandemic. This likely reflects higher self-reported symptom prevalence, as not all individuals seek medical care for their symptoms. Additionally, GPs may record an underlying condition rather than individual symptoms, leading to discrepancies between self-reports and medical records.

### Implications of the study

Given the occurrence of health symptoms with COVID-19 infection, and previously with infections such as Q-fever, and Lyme disease, monitoring symptomatology, in different organ systems, in the context of future outbreak of infections, could provide important information on the population health status and have a prognostic value for ill health. The significant differences between the three study groups in this current study, and also in Hampshire et al. [24], imply that future studies on similar outbreaks might want to look at symptom differences between people with an acute infection, with chronic symptoms, and non-infected people. Monitoring these differences from the acute infection onwards, rather than retrospectively, could improve understanding of the transition from acute to chronic illness, shed light on symptom trajectories, and might help to identify those at higher risk of long-term conditions, ideally at an early stage. This approach is especially relevant for new studies on post-COVID-19, since a vaccine is available, and any determining risk factors for who develops post-COVID-19 condition might be a strong argument for vaccinations for those risk groups. Also, by monitoring risk groups with new viral outbreaks, it is likely possible to respond to longer lasting effects early on, possibly increasing chances of better or full recovery.

For future research in this field, it would be valuable to include pre-existing psychological or chronic factors within and between group differences. In addition, inclusion of vaccination status as a confounding variable would be valuable, since this could affect (severity of) symptoms experienced. For this study, this was not possible since the questionnaire did not ask about vaccination status. Beyond focusing solely on chronic diseases, future research should consider the spectrum of symptoms affecting population health. After all, conditions are by definition a combination of enduring symptoms that are not as homogenous as they may seem. Finally, the Symptom and Perceptions questionnaire appeared to be an indispensable instrument in highlighting the importance of studying symptoms.

## Supporting information

S1 File **S1 Table. Incidence Rate Ratios for the post-covid versus the infected group on the SaP symptom variables (CI = 99%).** S2 Table**. Incidence Rate Ratios for the post-covid versus the non-infected group on the SaP symptom variables (CI = 99%).** S3 Table**. Incidence Rate Ratios for the infected versus the non-infected group on the SaP symptom variables (CI = 99%).** S4 Table**. Incidence rate ratios for the post-COVID-19 versus ex-covid including events during the pandemic.** Adjusted for age, gender, income, education, migration status, obesity, smoking behaviour, and excessive use of alcohol. S5 Table**. Incidence rate ratios for post-COVID-19 versus non-infected including events during the pandemic.** Adjusted for age, gender, income, education, migration status, obesity, smoking behaviour, and excessive use of alcohol. S6 Table**. Incidence rate ratios for ex-covid versus non-infected including events during the pandemic.** Adjusted for age, gender, income, education, migration status, obesity, smoking behaviour, and excessive use of alcohol.
(ZIP)
