# Supplementary material for: Health symptoms and post-COVID-19: Comparing symptomatic groups based on self-reported and primary care data
Source: PLoS One. 2025 Jun 12;20(6):e0323960. doi: 10.1371/journal.pone.0323960 (PMC12161569; doi:10.1371/journal.pone.0323960)
Supplement: S1 File — S1 Table. Incidence Rate Ratios for the post-covid versus the infected group on the SaP symptom variables (CI = 99%). S2 Table. Incidence Rate Ratios for the post-covid versus the non-infected group on the SaP symptom variables (CI = 99%). S3 Table. Incidence Rate Ratios for the infected versus the non-infected group on the SaP symptom variables (CI = 99%). S4 Table. Incidence rate ratios for the post-COVID-19 versus ex-covid including events during the pandemic. Adjusted for age, gender, income, education, migration status, obesity, smoking behaviour, and excessive use of alcohol. S5 Table. Incidence rate ratios for post-COVID-19 versus non-infected including events during the pandemic. Adjusted for age, gender, income, education, migration status, obesity, smoking behaviour, and excessive use of alcohol. S6 Table. Incidence rate ratios for ex-covid versus non-infected including events during the pandemic. Adjusted for age, gender, income, education, migration status, obesity, smoking behaviour, and excessive use of alcohol. (ZIP) [file pone.0323960.s001.zip › Supporting Information File_S1.docx]

**Supporting Information**

| **S1 Table. Incidence Rate Ratios for the post-covid versus the infected group on the SaP symptom variables (CI = 99%).** | | | | | | | | | | | | | | |
| --- | --- | --- | --- | --- | --- | --- | --- | --- | --- | --- | --- | --- | --- | --- |
|  | Number of symptoms | | | |  | Duration of symptoms | | | |  | Symptom severity | | | |
|  | IRR | CI | | |  | IRR | CI | | |  | IRR | CI | | |
| Post-covid group | **1.48** | **(1.46** | **-** | **1.49)** |  | **1.92** | **(1.88** | **-** | **1.96)** |  | **2.00** | **(1.96** | **-** | **2.05)** |
|  |  |  |  |  |  |  |  |  |  |  |  |  |  |  |
| Age | **.99** | **(.99** | **-** | **.99)** |  | **1.00** | **(1.00** | **-** | **1.00)** |  | **1.01** | **(1.00** | **-** | **1.01)** |
| Gender | **1.25** | **(1.24** | **-** | **1.26)** |  | **1.45** | **(1.42** | **-** | **1.47)** |  | **1.27** | **(1.25** | **-** | **1.29)** |
| Income (group 1 = reference) |  |  |  |  |  |  |  |  |  |  |  |  |  |  |
| Income group 2 | **.93** | **(.92** | **-** | **.94)** |  | **.84** | **(.81** | **-** | **.86)** |  | **.86** | **(.83** | **-** | **.88)** |
| Income group 3 | **.88** | **(.86** | **-** | **.89)** |  | **.76** | **(.74** | **-** | **.78)** |  | **.75** | **(.73** | **-** | **.76)** |
| Income group 4 | **.84** | **(.83** | **-** | **.86)** |  | **.71** | **(.69** | **-** | **.73)** |  | **.69** | **(.67** | **-** | **.71)** |
| Income group 5 | **.79** | **(.78** | **-** | **.80)** |  | **.65** | **(.63** | **-** | **.67)** |  | **.62** | **(.60** | **-** | **.64)** |
| Education (lower = reference) |  |  |  |  |  |  |  |  |  |  |  |  |  |  |
| Middle education | **1.03** | **(1.02** | **-** | **1.04)** |  | **1.03** | **(1.01** | **-** | **1.05)** |  | **.90** | **(.88** | **-** | **.92)** |
| Higher education | **1.01** | **(1.00** | **-** | **1.02)** |  | **.95** | **(.95** | **-** | **.96)** |  | **.78** | **(.77** | **-** | **.80)** |
| Migrational background (no migration = reference) |  |  |  |  |  |  |  |  |  |  |  |  |  |  |
| European migrant | **1.10** | **(1.09** | **-** | **1.12)** |  | **1.20** | **(1.16** | **-** | **1.23)** |  | **1.27** | **(1.23** | **-** | **1.31)** |
| Non-European migrant | **1.12** | **(1.10** | **-** | **1.13)** |  | **1.22** | **(1.19** | **-** | **1.25)** |  | **1.46** | **(1.43** | **-** | **1.50)** |
| Obesity | **1.15** | **(1.14** | **-** | **1.17)** |  | **1.29** | **(1.26** | **-** | **1.31)** |  | **1.31** | **(1.29** | **-** | **1.34)** |
| Smoking (non-smoker = reference) |  |  |  |  |  |  |  |  |  |  |  |  |  |  |
| Ex-smoker | **1.09** | **(1.08** | **-** | **1.10)** |  | **1.14** | **(1.13** | **-** | **1.16)** |  | **1.14** | **(1.12** | **-** | **1.16)** |
| Smoker | **1.16** | **(1.14** | **-** | **1.17)** |  | **1.25** | **(1.23** | **-** | **1.28)** |  | **1.27** | **(1.24** | **-** | **1.30)** |
| Excessive use of alcohol | **1.00** | **(.99** | **-** | **1.01)** |  | **.96** | **(.95** | **-** | **.98)** |  | **.92** | **(.91** | **-** | **.95)** |
|  |  |  |  |  |  |  |  |  |  |  |  |  |  |  |
| Constante | **8.70** | **(8.50** | **-** | **8.90)** |  | **1.49** | **(1.43** | **-** | **1.56)** |  | **1.20** | **(1.15** | **-** | **1.26)** |
