# Supplementary material for: Health symptoms and post-COVID-19: Comparing symptomatic groups based on self-reported and primary care data
Source: PLoS One. 2025 Jun 12;20(6):e0323960. doi: 10.1371/journal.pone.0323960 (PMC12161569; doi:10.1371/journal.pone.0323960)
Supplement: S1 File — S1 Table. Incidence Rate Ratios for the post-covid versus the infected group on the SaP symptom variables (CI = 99%). S2 Table. Incidence Rate Ratios for the post-covid versus the non-infected group on the SaP symptom variables (CI = 99%). S3 Table. Incidence Rate Ratios for the infected versus the non-infected group on the SaP symptom variables (CI = 99%). S4 Table. Incidence rate ratios for the post-COVID-19 versus ex-covid including events during the pandemic. Adjusted for age, gender, income, education, migration status, obesity, smoking behaviour, and excessive use of alcohol. S5 Table. Incidence rate ratios for post-COVID-19 versus non-infected including events during the pandemic. Adjusted for age, gender, income, education, migration status, obesity, smoking behaviour, and excessive use of alcohol. S6 Table. Incidence rate ratios for ex-covid versus non-infected including events during the pandemic. Adjusted for age, gender, income, education, migration status, obesity, smoking behaviour, and excessive use of alcohol. (ZIP) [file pone.0323960.s001.zip › Supporting Information file_S2.docx]

**Supporting Information**

| **S2 Table. Incidence Rate Ratios for the post-covid versus the non-infected group on the SaP symptom variables (CI = 99%).** | | | | | | | | | | | | | | |
| --- | --- | --- | --- | --- | --- | --- | --- | --- | --- | --- | --- | --- | --- | --- |
|  | Number of symptoms | | | |  | Duration of symptoms | | | |  | Symptom severity | | | |
|  | IRR | CI | | |  | IRR | CI | | |  | IRR | CI | | |
| Post-covid group | **1.55** | **(1.52** | **-** | **1.57)** |  | **1.87** | **(1.82** | **-** | **1.92)** |  | **1.95** | **(1.89** | **-** | **2.01)** |
|  |  |  |  |  |  |  |  |  |  |  |  |  |  |  |
| Age | **.99** | **(.99** | **-** | **.99)** |  | **1.00** | **(1.00** | **-** | **1.00)** |  | **1.00** | **(1.00** | **-** | **1.01)** |
| Gender | **1.26** | **(1.25** | **-** | **1.27)** |  | **1.43** | **(1.40** | **-** | **1.45)** |  | **1.25** | **(1.23** | **-** | **1.28)** |
| Income (group 1 = reference) |  |  |  |  |  |  |  |  |  |  |  |  |  |  |
| Income group 2 | **.91** | **(.90** | **-** | **.93)** |  | **.82** | **(.79** | **-** | **.85)** |  | **.84** | **(.81** | **-** | **.87)** |
| Income group 3 | **.86** | **(.84** | **-** | **.88)** |  | **.74** | **(.72** | **-** | **.77)** |  | **.73** | **(.70** | **-** | **.76)** |
| Income group 4 | **.84** | **(.82** | **-** | **.85)** |  | **.71** | **(.68** | **-** | **.73)** |  | **.68** | **(.66** | **-** | **.71)** |
| Income group 5 | **.78** | **(.76** | **-** | **.80)** |  | **.64** | **(.62** | **-** | **.67)** |  | **.61** | **(.58** | **-** | **.63)** |
| Education (lower = reference) |  |  |  |  |  |  |  |  |  |  |  |  |  |  |
| Middle education | **1.06** | **(1.05** | **-** | **1.08)** |  | **1.07** | **(1.05** | **-** | **1.10)** |  | **.95** | **(.93** | **-** | **.98)** |
| Higher education | **1.07** | **(1.05** | **-** | **1.08)** |  | **1.05** | **(1.03** | **-** | **1.08)** |  | **.85** | **(.83** | **-** | **.88)** |
| Migrational background (no migration = reference) |  |  |  |  |  |  |  |  |  |  |  |  |  |  |
| European migrant | **1.08** | **(1.06** | **-** | **1.11)** |  | **1.12** | **(1.07** | **-** | **1.16)** |  | **1.19** | **(1.14** | **-** | **1.24)** |
| Non-European migrant | **1.09** | **(1.07** | **-** | **1.11)** |  | **1.12** | **(1.09** | **-** | **1.16)** |  | **1.38** | **(1.33** | **-** | **1.43)** |
| Obesity | **1.18** | **(1.16** | **-** | **1.20)** |  | **1.31** | **(1.28** | **-** | **1.34)** |  | **1.30** | **(1.27** | **-** | **1.34)** |
| Smoking (non-smoker = reference) |  |  |  |  |  |  |  |  |  |  |  |  |  |  |
| Ex-smoker | **1.10** | **(1.09** | **-** | **1.12)** |  | **1.15** | **(1.13** | **-** | **1.18)** |  | **1.13** | **(1.11** | **-** | **1.16)** |
| Smoker | **1.14** | **(1.12** | **-** | **1.16)** |  | **1.26** | **(1.23** | **-** | **1.30)** |  | **1.24** | **(1.20** | **-** | **1.28)** |
| Excessive use of alcohol | **.97** | **(.96** | **-** | **.99)** |  | **.95** | **(.92** | **-** | **.97)** |  | **.90** | **(.87** | **-** | **.92)** |
|  |  |  |  |  |  |  |  |  |  |  |  |  |  |  |
| Constante | **7.59** | **(7.31** | **-** | **7.87)** |  | **1.59** | **(1.50** | **-** | **1.70)** |  | **1.28** | **(1.20** | **-** | **1.38)** |
