# Supplementary material for: Health symptoms and post-COVID-19: Comparing symptomatic groups based on self-reported and primary care data
Source: PLoS One. 2025 Jun 12;20(6):e0323960. doi: 10.1371/journal.pone.0323960 (PMC12161569; doi:10.1371/journal.pone.0323960)
Supplement: S1 File — S1 Table. Incidence Rate Ratios for the post-covid versus the infected group on the SaP symptom variables (CI = 99%). S2 Table. Incidence Rate Ratios for the post-covid versus the non-infected group on the SaP symptom variables (CI = 99%). S3 Table. Incidence Rate Ratios for the infected versus the non-infected group on the SaP symptom variables (CI = 99%). S4 Table. Incidence rate ratios for the post-COVID-19 versus ex-covid including events during the pandemic. Adjusted for age, gender, income, education, migration status, obesity, smoking behaviour, and excessive use of alcohol. S5 Table. Incidence rate ratios for post-COVID-19 versus non-infected including events during the pandemic. Adjusted for age, gender, income, education, migration status, obesity, smoking behaviour, and excessive use of alcohol. S6 Table. Incidence rate ratios for ex-covid versus non-infected including events during the pandemic. Adjusted for age, gender, income, education, migration status, obesity, smoking behaviour, and excessive use of alcohol. (ZIP) [file pone.0323960.s001.zip › Supporting Information file_S3.docx]

**Supporting Information**

| **S3 Table. Incidence Rate Ratios for the infected versus the non-infected group on the SaP symptom variables (CI = 99%).** | | | | | | | | | | | | | | |
| --- | --- | --- | --- | --- | --- | --- | --- | --- | --- | --- | --- | --- | --- | --- |
|  | Number of Symptoms | | | |  | Duration of symptoms | | | |  | Severity of symptoms | | | |
|  | IRR | CI | | |  | IRR | CI | | |  | IRR | CI | | |
| Infected | **1.04** | **(1.03** | **-** | **1.05)** |  | **.98** | **(.96** | **-** | **.99)** |  | **.98** | **(.97** | **-** | **1.00)** |
|  |  |  |  |  |  |  |  |  |  |  |  |  |  |  |
| Age | **.99** | **(.99** | **-** | **.99)** |  | **1.00** | **(1.00** | **-** | **1.00)** |  | **1.01** | **(1.01** | **-** | **1.01)** |
| Gender | **1.27** | **(1.26** | **-** | **1.28)** |  | **1.45** | **(1.43** | **-** | **1.47)** |  | **1.27** | **(1.25** | **-** | **1.29)** |
| Income (group 1 = reference) |  |  |  |  |  |  |  |  |  |  |  |  |  |  |
| Income group 2 | **.92** | **(.91** | **-** | **.93)** |  | **.82** | **(.79** | **-** | **.84)** |  | **.84** | **(.81** | **-** | **.86)** |
| Income group 3 | **.86** | **(.85** | **-** | **.88)** |  | **.74** | **(.72** | **-** | **.76)** |  | **.73** | **(.71** | **-** | **.75)** |
| Income group 4 | **.83** | **(.82** | **-** | **.85)** |  | **.70** | **(.68** | **-** | **.72)** |  | **.68** | **(.66** | **-** | **.70)** |
| Income group 5 | **.77** | **(.77** | **-** | **.79)** |  | **.64** | **(.62** | **-** | **.66)** |  | **.61** | **(.59** | **-** | **.62)** |
| Education (lower = reference) |  |  |  |  |  |  |  |  |  |  |  |  |  |  |
| Middle education | **1.06** | **(1.05** | **-** | **1.07)** |  | **1.06** | **(1.04** | **-** | **1.08)** |  | **.93** | **(.92** | **-** | **.95)** |
| Higher education | **1.04** | **(1.03** | **-** | **1.05)** |  | **1.01** | **(.99** | **-** | **1.03)** |  | **.82** | **(.80** | **-** | **.83)** |
| Migrational background (no migration = reference) |  |  |  |  |  |  |  |  |  |  |  |  |  |  |
| European migrant | **1.09** | **(1.07** | **-** | **1.11)** |  | **1.16** | **(1.13** | **-** | **1.19)** |  | **1.24** | **(1.20** | **-** | **1.28)** |
| Non-European migrant | **1.10** | **(1.08** | **-** | **1.11)** |  | **1.16** | **(1.13** | **-** | **1.19)** |  | **1.42** | **(1.38** | **-** | **1.46)** |
| Obesity | **1.18** | **(1.16** | **-** | **1.19)** |  | **1.32** | **(1.30** | **-** | **1.35)** |  | **1.32** | **(1.30** | **-** | **1.35)** |
| Smoking (non-smoker = reference) |  |  |  |  |  |  |  |  |  |  |  |  |  |  |
| Ex-smoker | **1.11** | **(1.10** | **-** | **1.12)** |  | **1.17** | **(1.50** | **-** | **1.19)** |  | **1.16** | **(1.14** | **-** | **1.18)** |
| Smoker | **1.16** | **(1.15** | **-** | **1.17)** |  | **1.28** | **(1.25** | **-** | **1.31)** |  | **1.28** | **(1.25** | **-** | **1.32)** |
| Excessive use of alcohol | **.98** | **(.97** | **-** | **.99)** |  | **.95** | **(.93** | **-** | **.97)** |  | **.91** | **(.90** | **-** | **.93)** |
|  |  |  |  |  |  |  |  |  |  |  |  |  |  |  |
| Constante | **7.92** | **(7.71** | **-** | **8.13)** |  | **1.49** | **(1.42** | **-** | **1.56)** |  | **1.20** | **(1.13** | **-** | **1.26)** |
