# Supplementary material for: Health symptoms and post-COVID-19: Comparing symptomatic groups based on self-reported and primary care data
Source: PLoS One. 2025 Jun 12;20(6):e0323960. doi: 10.1371/journal.pone.0323960 (PMC12161569; doi:10.1371/journal.pone.0323960)
Supplement: S1 File — S1 Table. Incidence Rate Ratios for the post-covid versus the infected group on the SaP symptom variables (CI = 99%). S2 Table. Incidence Rate Ratios for the post-covid versus the non-infected group on the SaP symptom variables (CI = 99%). S3 Table. Incidence Rate Ratios for the infected versus the non-infected group on the SaP symptom variables (CI = 99%). S4 Table. Incidence rate ratios for the post-COVID-19 versus ex-covid including events during the pandemic. Adjusted for age, gender, income, education, migration status, obesity, smoking behaviour, and excessive use of alcohol. S5 Table. Incidence rate ratios for post-COVID-19 versus non-infected including events during the pandemic. Adjusted for age, gender, income, education, migration status, obesity, smoking behaviour, and excessive use of alcohol. S6 Table. Incidence rate ratios for ex-covid versus non-infected including events during the pandemic. Adjusted for age, gender, income, education, migration status, obesity, smoking behaviour, and excessive use of alcohol. (ZIP) [file pone.0323960.s001.zip › Supporting Information file_S4.docx]

**Supporting Information**

| **S4 Table. Incidence rate ratios for the post-COVID-19 versus ex-covid including events during the pandemic.** Adjusted for age, gender, income, education, migration status, obesity, smoking behaviour, and excessive use of alcohol | | | | | | | | | | | | | | |
| --- | --- | --- | --- | --- | --- | --- | --- | --- | --- | --- | --- | --- | --- | --- |
|  | Number of symptoms | | | |  | Symptom duration | | | | | Symptom severity | | | |
|  | IRR | CI | | |  | IRR | CI | | |  | IRR | CI | | |
| Post-covid vs Ex-covid | **1.48** | **(1.46** | **-** | **1.49)** |  | **1.91** | **(1.87** | **-** | **1.95)** |  | **1.99** | **(1.95** | **-** | **2.04)** |
| I personally experienced hospitalization due to COVID-19 | **1.16** | **(1.12** | **-** | **1.20)** |  | **1.33** | **(1.25** | **-** | **1.42)** |  | **1.60** | **(1.50** | **-** | **1.71)** |
| Group * Event | **.89** | **(.85** | **-** | **.93)** |  | **.85** | **(.77** | **-** | **.93)** |  | **.73** | **(.66** | **-** | **.81)** |
|  |  |  |  |  |  |  |  |  |  |  |  |  |  |  |
| Post-covid vs Ex-covid | **1.47** | **(1.46** | **-** | **1.49)** |  | **1.91** | **(1.87** | **-** | **1.96)** |  | **1.98** | **(1.93** | **-** | **2.03)** |
| Someone significant to me was hospitalized due to COVID-19 | **1.12** | **(1.11** | **-** | **1.14)** |  | **1.18** | **(1.15** | **-** | **1.20)** |  | **1.25** | **(1.23** | **-** | **1.28)** |
| Group * Event | .97 | (.94 | - | 1.00) |  | .97 | (.92 | - | 1.02) |  | .98 | (.93 | - | 1.04) |
|  |  |  |  |  |  |  |  |  |  |  |  |  |  |  |
| Post-covid vs Ex-covid | **1.48** | **(1.46** | **-** | **1.49)** |  | **1.92** | **(1.87** | **-** | **1.96)** |  | **2.00** | **(1.95** | **-** | **2.04)** |
| Someone significant to me passed away due to COVID-19 | **1.12** | **(1.11** | **-** | **1.14)** |  | **1.19** | **(1.16** | **-** | **1.22)** |  | **1.28** | **(1.24** | **-** | **1.31)** |
| Group * Event | .97 | (.94 | - | 1.01) |  | .97 | (.92 | - | 1.03) |  | .95 | (.89 | - | 1.02) |
|  |  |  |  |  |  |  |  |  |  |  |  |  |  |  |
| Post-covid vs Ex-covid | **.68** | **(.67** | **-** | **.69)** |  | **1.92** | **(1.88** | **-** | **1.97)** |  | **2.00** | **(1.95** | **-** | **2.05)** |
| Due to the COVID-19 social distancing measures, I could not say goodbye to someone significant to me | **1.13** | **(1.10** | **-** | **1.16)** |  | **1.22** | **(1.19** | **-** | **1.24)** |  | **1.25** | **(1.22** | **-** | **1.28)** |
| Group * Event | 1.02 | (.99 | - | 1.05) |  | .95 | (.90 | - | 1.01) |  | .96 | (.90 | - | 1.02) |
