# Supplementary material for: Health symptoms and post-COVID-19: Comparing symptomatic groups based on self-reported and primary care data
Source: PLoS One. 2025 Jun 12;20(6):e0323960. doi: 10.1371/journal.pone.0323960 (PMC12161569; doi:10.1371/journal.pone.0323960)
Supplement: S1 File — S1 Table. Incidence Rate Ratios for the post-covid versus the infected group on the SaP symptom variables (CI = 99%). S2 Table. Incidence Rate Ratios for the post-covid versus the non-infected group on the SaP symptom variables (CI = 99%). S3 Table. Incidence Rate Ratios for the infected versus the non-infected group on the SaP symptom variables (CI = 99%). S4 Table. Incidence rate ratios for the post-COVID-19 versus ex-covid including events during the pandemic. Adjusted for age, gender, income, education, migration status, obesity, smoking behaviour, and excessive use of alcohol. S5 Table. Incidence rate ratios for post-COVID-19 versus non-infected including events during the pandemic. Adjusted for age, gender, income, education, migration status, obesity, smoking behaviour, and excessive use of alcohol. S6 Table. Incidence rate ratios for ex-covid versus non-infected including events during the pandemic. Adjusted for age, gender, income, education, migration status, obesity, smoking behaviour, and excessive use of alcohol. (ZIP) [file pone.0323960.s001.zip › Supporting Information File_S5.docx]

**Supporting Information**

| **S5 Table. Incidence rate ratios for post-COVID-19 versus non-infected including events during the pandemic.**  Adjusted for age, gender, income, education, migration status, obesity, smoking behaviour, and excessive use of alcohol | | | | | | | | | | | | | | |
| --- | --- | --- | --- | --- | --- | --- | --- | --- | --- | --- | --- | --- | --- | --- |
|  | Number of symptoms | | | |  | Symptom duration | | | | | Symptom severity | | | |
|  | IRR | CI | | |  | IRR | CI | | |  | IRR | CI | | |
| Post-covid versus non-infected | **1.54** | **(1.52** | **-** | **1.56)** |  | **1.84** | **(1.80** | **-** | **1.88)** |  | **1.94** | **(1.89** | **-** | **1.99)** |
| I personally experienced hospitalization due to COVID-19 | **1.18** | **(1.11** | **-** | **1.26)** |  | **1.28** | **(1.15** | **-** | **1.42)** |  | **1.62** | **(1.44** | **-** | **1.82)** |
| Group * Event | **.86** | **(.80** | **-** | **.93)** |  | .90 | (.79 | - | 1.02) |  | **.73** | **(.64** | **-** | **.84)** |
|  |  |  |  |  |  |  |  |  |  |  |  |  |  |  |
| Post-covid versus non-infected | **1.55** | **(1.53** | **-** | **1.57)** |  | **1.84** | **(1.80** | **-** | **1.89)** |  | **1.94** | **(1.89** | **-** | **1.99)** |
| Someone significant to me was hospitalized due to COVID-19 | **1.18** | **(1.16** | **-** | **1.19)** |  | **1.20** | **(1.17** | **-** | **1.23)** |  | **1.29** | **(1.26** | **-** | **1.33)** |
| Group * Event | **.93** | **(.90** | **-** | **.96)** |  | .96 | (.91 | - | 1.01) |  | .96 | (.90 | - | 1.01) |
|  |  |  |  |  |  |  |  |  |  |  |  |  |  |  |
| Post-covid versus non-infected | **1.55** | **(1.52** | **-** | **1.57)** |  | **1.85** | **(1.81** | **-** | **1.89)** |  | **1.95** | **(1.90** | **-** | **2.00)** |
| Someone significant to me passed away due to COVID-19 | **1.17** | **(1.15** | **-** | **1.19)** |  | **1.21** | **(1.18** | **-** | **1.24)** |  | **1.31** | **(1.27** | **-** | **1.35)** |
| Group * Event | **.94** | **(.90** | **-** | **.97)** |  | .97 | (.91 | - | 1.03) |  | .94 | (.88 | - | 1.00) |
|  |  |  |  |  |  |  |  |  |  |  |  |  |  |  |
| Post-covid versus non-infected | **1.55** | **(1.53** | **-** | **1.57)** |  | **1.87** | **(1.82** | **-** | **1.91)** |  | **1.96** | **(1.91** | **-** | **2.01)** |
| Due to the COVID-19 social distancing measures, I could not say goodbye to someone significant to me | **1.18** | **(1.17** | **-** | **1.20)** |  | **1.24** | **(1.21** | **-** | **1.27)** |  | **1.28** | **(1.25** | **-** | **1.31)** |
| Group * Event | **.95** | **(.92** | **-** | **.98)** |  | **.94** | **(.89** | **-** | **1.00)** |  | .95 | (.89 | - | 1.01) |
|  | | | | | | | | | | | | | | |
