# Supplementary material for: Health symptoms and post-COVID-19: Comparing symptomatic groups based on self-reported and primary care data
Source: PLoS One. 2025 Jun 12;20(6):e0323960. doi: 10.1371/journal.pone.0323960 (PMC12161569; doi:10.1371/journal.pone.0323960)
Supplement: S1 File — S1 Table. Incidence Rate Ratios for the post-covid versus the infected group on the SaP symptom variables (CI = 99%). S2 Table. Incidence Rate Ratios for the post-covid versus the non-infected group on the SaP symptom variables (CI = 99%). S3 Table. Incidence Rate Ratios for the infected versus the non-infected group on the SaP symptom variables (CI = 99%). S4 Table. Incidence rate ratios for the post-COVID-19 versus ex-covid including events during the pandemic. Adjusted for age, gender, income, education, migration status, obesity, smoking behaviour, and excessive use of alcohol. S5 Table. Incidence rate ratios for post-COVID-19 versus non-infected including events during the pandemic. Adjusted for age, gender, income, education, migration status, obesity, smoking behaviour, and excessive use of alcohol. S6 Table. Incidence rate ratios for ex-covid versus non-infected including events during the pandemic. Adjusted for age, gender, income, education, migration status, obesity, smoking behaviour, and excessive use of alcohol. (ZIP) [file pone.0323960.s001.zip › Supporting Information file_S6.docx]

**Supporting Information**

| **S6 Table. Incidence rate ratios for ex-covid versus non-infected including events during the pandemic.** Adjusted for age, gender, income, education, migration status, obesity, smoking behaviour, and excessive use of alcohol | | | | | | | | | | | | | | |
| --- | --- | --- | --- | --- | --- | --- | --- | --- | --- | --- | --- | --- | --- | --- |
|  | Number of symptoms | | | |  | Symptom duration | | | | | Symptom severity | | | |
|  | IRR | CI | | |  | IRR | CI | | |  | IRR | CI | | |
| Ex-covid versus non-infected | **1.04** | **(1.03** | **-** | **1.04)** |  | **.97** | **(.96** | **-** | **.98)** |  | **.98** | **(.97** | **-** | **.99)** |
| I personally experienced hospitalization due to COVID-19 | **1.21** | **(1.14** | **-** | **1.28)** |  | **1.29** | **(1.16** | **-** | **1.44)** |  | **1.67** | **(1.49** | **-** | **1.87)** |
| Group * Event | .96 | (.90 | **-** | 1.03) |  | 1.04 | (.91 | **-** | 1.18) |  | .96 | (.84 | **-** | 1.10) |
|  |  |  |  |  |  |  |  |  |  |  |  |  | **-** |  |
| Ex-covid versus non-infected | **1.04** | **(1.04** | **-** | **1.05)** |  | **.97** | **(.96** | **-** | **.98)** |  | **.99** | **(.97** | **-** | **1.00)** |
| Someone significant to me was hospitalized due to COVID-19 | **1.18** | **(1.16** | **-** | **1.19)** |  | **1.20** | **(1.17** | **-** | **1.23)** |  | **1.29** | **(1.26** | **-** | **1.33)** |
| Group * Event | **.96** | **(.94** | **-** | **.97)** |  | .99 | (.95 | **-** | 1.02) |  | .97 | (.94 | - | 1.01) |
|  |  |  |  |  |  |  |  |  |  |  |  |  | **-** |  |
| Ex-covid versus non-infected | **1.04** | **(1.04** | **-** | **1.05)** |  | **.97** | **(.96** | **-** | **.99)** |  | **.99** | **(.97** | **-** | **1.00)** |
| Someone significant to me passed away due to COVID-19 | **1.17** | **(1.15** | **-** | **1.19)** |  | **1.21** | **(1.17** | **-** | **1.24)** |  | **1.31** | **(1.27** | **-** | **1.35)** |
| Group * Event | **.96** | **(.94** | **-** | **.98)** |  | .99 | (.95 | - | 1.03) |  | .98 | (.94 | - | 1.02) |
|  |  |  |  |  |  |  |  |  |  |  |  |  | **-** |  |
| Ex-covid versus non-infected | **1.04** | **(1.04** | **-** | **1.05)** |  | **.98** | **(.97** | **-** | **.99)** |  | **.99** | **(.98** | **-** | **1.00)** |
| Due to the COVID-19 social distancing measures, I could not say goodbye to someone significant to me | **1.19** | **(1.17** | **-** | **1.20)** |  | **1.24** | **(1.21** | **-** | **1.26)** |  | **1.28** | **(1.25** | **-** | **1.31)** |
| Group * Event | **.97** | **(.95** | **-** | **.98)** |  | .99 | (.95 | - | 1.02) |  | .98 | (.95 | - | 1.01) |
